# Empiric tuberculosis treatment and 12-month mortality among sputum GeneXpert-negative adults living with HIV in Uganda in the era of widespread antiretroviral therapy: A prospective cohort study

**DOI:** 10.1371/journal.pone.0347436

**Published:** 2026-09-08

**Authors:** Lydia Nakiyingi, Bernard Kikaire, Sarah Nakayenga, Louis Kamulegeya, Elizabeth Nakabugo, Juliet Nkugwa Asio, Bernard Bagaya, Willy Ssengooba, Harriet Mayanja-Kizza, Yukari C. Manabe

**Affiliations:** 1 Department of Medicine, School of Medicine, Makerere University College of Health Sciences, Kampala, Uganda; 2 Makerere University Biomedical Research Centre, Makerere University College of Health Sciences, Kampala, Uganda; 3 Infectious Diseases Institute, Makerere University College of Health Sciences, Kampala, Uganda; 4 Department of Pediatrics and Child Health, School of Medicine, College of Health Sciences, Makerere University, Kampala, Uganda; 5 Department of General Virology, Uganda Virus Research Institute, Entebbe, Uganda; 6 Department of Immunology and Molecular Biology, School of Biomedical Sciences, College of Health Sciences, Makerere University, Kampala, Uganda; 7 Division of Infectious Diseases, Johns Hopkins School of Medicine, Baltimore, United States of America; Rutgers Biomedical and Health Sciences, UNITED STATES OF AMERICA

## Abstract

**Background:**

In sub-Saharan Africa where both tuberculosis (TB) and HIV are prevalent, empiric TB treatment in people living with HIV (PLHIV) persists due to limited sensitivity of sputum-based TB tests. We evaluated mortality among molecular test-negative presumptive TB adult PLHIV in a population with widespread antiretroviral therapy (ART) coverage, comparing those empirically treated and not treated for TB.

**Materials and methods:**

From November 2017 to December 2020, Xpert-negative adult PLHIV were recruited at Mulago Referral Hospital and Kisenyi Health Centre-IV in Kampala, Uganda. Clinical data including TB symptoms, chest X-ray, and empiric TB treatment decision were collected. Laboratory investigations included CD4 cell count, serum cryptococcal antigen (CrAg), urine TB-lipoarabinomannan (TB-LAM), microbiological blood cultures, and sputum mycobacterial growth indicator tube (MGIT) cultures. Participants were followed monthly for 12 months to ascertain vital status.

**Results:**

Overall, 300 participants were enrolled; 61.3% inpatients, 55.7% female, median age 37 (IQR 29–45) years, 82.3% on ART, median CD4 206 cells/mm³ (IQR 36–507). Of the 300, 68 (22.7%) received empiric TB treatment, of which 53 (77.9%) were inpatients. 12-month mortality was 31.0% (93/300); 72% within three months post-enrolment. TB cultures were positive in 5.0% (15/300), 12.3% had positive CrAg, and 3.7% had positive blood culture. Empirically treated participants had higher mortality (42.7%) than non-treated (27.6%) although the difference was not statistically significant after adjusting for factors indicative of severe illness (adjusted Mortality Rate Ratio- aMRR 1.05, p = 0.850). Inpatient (aMRR 3.80, p = 0.008) and poor functional status (aMRR 3.5 95%, p = 0.001) were independently associated with 12-month mortality.

**Conclusion:**

We found high 12-month mortality among Xpert-negative PLHIV, predominantly inpatients and within three months post-enrolment. Although empiric TB treatment was associated with higher mortality, this association was lost after adjustment for severe illness indicators. Cryptococcal antigenemia and bacteremia were not uncommon, underscoring need for comprehensive evaluations for non-TB conditions alongside empiric TB treatment.

## Introduction

Tuberculosis (TB) remains the leading cause of death among people living with HIV (PLHIV) globally, accounting for approximately one in every three AIDS-related deaths [1,2]. According to the World Health Organization (WHO) Global Tuberculosis Report 2025 [3], approximately 150,000 HIV/TB co-infected people died in 2024. TB often goes undiagnosed because standard sputum-based diagnostic tools such as Xpert MTB/RIF (herein referred to as Xpert) and sputum smear microscopy, have limited sensitivity in PLHIV [4,5]. Postmortem studies in sub-Saharan Africa (SSA) report up to 46% of TB cases remain undetected antemortem [6,7].

Among PLHIV with advanced immunosuppression, clinicians in high-burden HIV/TB settings often resort to empiric TB treatment; initiating TB therapy without microbiological confirmation, including situations where the Xpert result is negative [8,9]. In Uganda, one study found that up to 30% of smear-negative presumptive TB PLHIV received empiric TB treatment [9]. The introduction of Xpert, a more sensitive PCR-based test for MTB detection in HIV/TB high-burden settings, raised expectations for more rapid and accurate TB diagnosis, with decrease in TB-associated morbidity and mortality [10–13]. However, a considerable number of PLHIV still test negative on these assays [14–16], leading to minimal improvements in outcomes. An important limitation of Xpert is its low performance in paucibacillary disease commonly seen in PLHIV [5,11,16]. Xpert Ultra has shown better sensitivity than Xpert MTB/RIF for smear-negative and paucibacillary [13,16] and may reduce reliance on empiric TB treatment in this population. However, at the time of this study, Xpert Ultra had not yet been introduced as a TB diagnostic at the study site.

Empiric TB treatment remains common in high HIV/TB burden countries often driven by diagnostic uncertainty, severe immunosuppression, clinical severity at presentation, and the desire to improve patient outcomes [9,17–19]. While current TB diagnosis and treatment guidelines [20] acknowledge the role of clinical judgement in the diagnosis of TB among PLHIV when microbiological confirmation is unavailable, empiric TB treatment carries the risk of unnecessary exposure to TB treatment, potential undesirable drug effects, and poor patient outcomes [15,21–23]. These challenges are accentuated by concerns of difficulty in monitoring TB treatment response among patients who receive empiric TB treatment as well as managing associated adverse drug reactions [24].

Despite limited data supporting the efficacy of empiric TB therapy [9,14,15,22,25,26], clinicians especially in low- and middle-income countries (LMICs) continue to rely on less sensitive diagnostic tools such as smear microscopy, chest X-ray (CXR), response to antibiotics, and clinical presentation to inform and guide their TB treatment decisions particularly among PLHIV [8,9,19,23]. Several factors drive empiric TB treatment in LMICs, most of which may not be readily modifiable [9,23]. The low CD4 cell counts commonly observed in this population exponentially increases the risk of TB infection, often tilting the balance toward empiric TB treatment in severely immunocompromised individuals [18,27]. The high rates of incident TB disease and mortality among TB tests-negative PLHIV who do not receive TB treatment after initial evaluation suggest potential survival benefits from empiric TB treatment [28–30].

Randomized controlled trials including REMEMBER [22] and STATIS [26] have demonstrated no mortality benefit from empiric TB treatment, particularly among patients not yet on antiretroviral therapy (ART). These trials were conducted under controlled research conditions with close monitoring. Real-world evidence from routine clinical care settings in high TB/HIV burden countries, where the majority of patients are on or have been on ART, and where advanced diagnostics are often unavailable, remains limited. We assessed 12-month mortality among sputum Xpert-negative presumptive TB adult PLHIV in Uganda, comparing those who received empiric TB treatment and those who did not, in a population where the majority are or have been on ART. The aim was to generate real-world data on the outcomes of empiric TB treatment in a high TB/HIV burden setting with high ART coverage. We also investigated concurrent or alterative non-TB conditions in this HIV population.

## Materials and methods

### Study design and setting

This was a prospective study among adult sputum Xpert-negative presumptive TB PLHIV recruited from Kisenyi Health Centre-IV (outpatients), a Kampala City Council Authority Clinic and Mulago-Kiruddu National Referral Hospital (MNRH) (inpatients) between 1^st^ November 2017–30^th^ December 2020. In Uganda, Xpert was introduced in PLHIV in 2010 at tertiary referral centers and was available as the main TB diagnostic at both facilities during the study period, Xpert Ultra had not yet been introduced. Other investigations for TB at the enrolment sites included chest X-ray (CXR) and smear microscopy. Both sputum TB culture and urine-TB lipoarabinomannan (TB-LAM) for PLHIV were only available for participants in this study.

All TB investigations and treatment at the facilities are free of charge. Patients diagnosed with active TB are treated in accordance with the existing treatment guidelines from the Uganda Ministry of Health National TB program [31].

### Patient recruitment and study population

PLHIV aged 18 years and above, clinically suspected to have active TB as per the WHO guidelines [20], who had undergone routine evaluations for pulmonary TB by the attending team were screened for eligibility. Eligible patients with confirmed HIV, suspected to have active TB based on the WHO criteria, and had received a negative Xpert test were enrolled into the study. Patients who had been initiated on anti-TB medication prior to the enrollment visit were excluded.

Sociodemographic and medical information including TB symptoms and signs, previous TB treatment, history of antiretroviral therapy (ART) was obtained from consenting participants. Information on clinical decision made by the attending clinician regarding empiric TB treatment initiation was obtained from participants’ medical records. Participants provided one spot sputum sample for mycobacterial growth indicator tube (MGIT) TB culture. Blood was collected for microbiological blood cultures, CD4 cell count and serum Cryptococcal Antigen (CrAg) (IMMY CrAg® LFA, Norman, Oklahoma, USA). Each participant provided a urine sample for TB-LAM testing (TB-LAM Alere, Waltham, MA, USA).

CXR findings interpreted by the attending clinician were obtained from participant records. Participants had monthly telephone follow-ups for 12 months post-enrolment to obtain data on survival status recorded as either dead or alive. For participants who died while in hospital, the date of death (DOD) was recorded immediately, while for those who died after discharge, the DOD was recorded during the telephone follow-up call. However, if the exact DOD was not available, the date of the telephone follow-up was captured as the DOD.

All laboratory procedures were done following standard laboratory procedures at the Mycobacteriology (BSL-3) Laboratory at the Department of Medical Microbiology, Makerere University (College of American Pathologists (CAP: ISO15189) Accredited Laboratory). A participant was considered TB culture positive if MGIT culture was positive, while positive blood culture was defined as the isolation of organisms (bacterial or fungal) from blood culture media. For the TB-LAM test, results were graded from 1+ to 4 + .

All study laboratory results were made available to the attending clinicians. Discharged participants were contacted by telephone to deliver results and those whose tests were positive were requested to return for treatment. Participants whose results were positive but could not be contacted by telephone had study home visits performed during which information on any medical treatment, and survival status were obtained.

### Statistical analysis

The primary outcome was survival status at 12 months post-enrolment among presumptive TB PLHIV with negative Xpert test, stratified by empiric TB treatment status. Continuous variables were summarized using medians and inter-quartile ranges (IQR) while categorical variables using proportions. Study population characteristics stratified by empiric TB treatment status were compared using chi-square tests for categorical variables and the Mann-Whitney U test for continuous variables. Kaplan-Meier survival analysis was used to estimate time-to-death, and survival probabilities with 95% confidence intervals (95%CI). The log-rank test was used to compare survival curves across different subgroups including empiric TB treatment status and inpatient versus outpatient status. Mortality rates were calculated per 1,000 person-months of follow-up, and differences between groups were assessed using mortality rate ratios (MRR) with corresponding 95% CI. Person-time at risk was calculated from the date of enrolment to the earliest time of death, loss to follow-up, or end of the 12-month observation period. Poisson regression with robust standard errors was used to identify independent predictors of mortality. To address potential confounding by indication that could have resulted from the difference in illness severity between participants who received empiric TB treatment and those who did not, illness severity markers were retained in the multivariable model regardless of statistical significance at bivariate analysis. The illness severity markers included CD4 cell count, body mass index (BMI), Karnofsky Performance Score (KPS), and inpatient status, together with ART use and age. Additional variables associated with mortality at a p-value < 0.20 in the bivariate analysis were also included. Mortality rate ratios (MRRs) with 95% CI were reported. All data were analyzed using STATA® version 18.0 (StataCorp, 4905 Lakeway Drive College Station, Texas USA).

### Ethical considerations

The study was approved by the Mulago Hospital Research and Ethics Committee (MHREC# 1120) Kampala, Uganda and the Uganda National Council for Science and Technology (UNCST#HS72ES). All participants provided written informed consent before participation in the study. All consent forms were approved by the above-mentioned ethics committees.

## Results

### Participant characteristics

**Fig 1:**
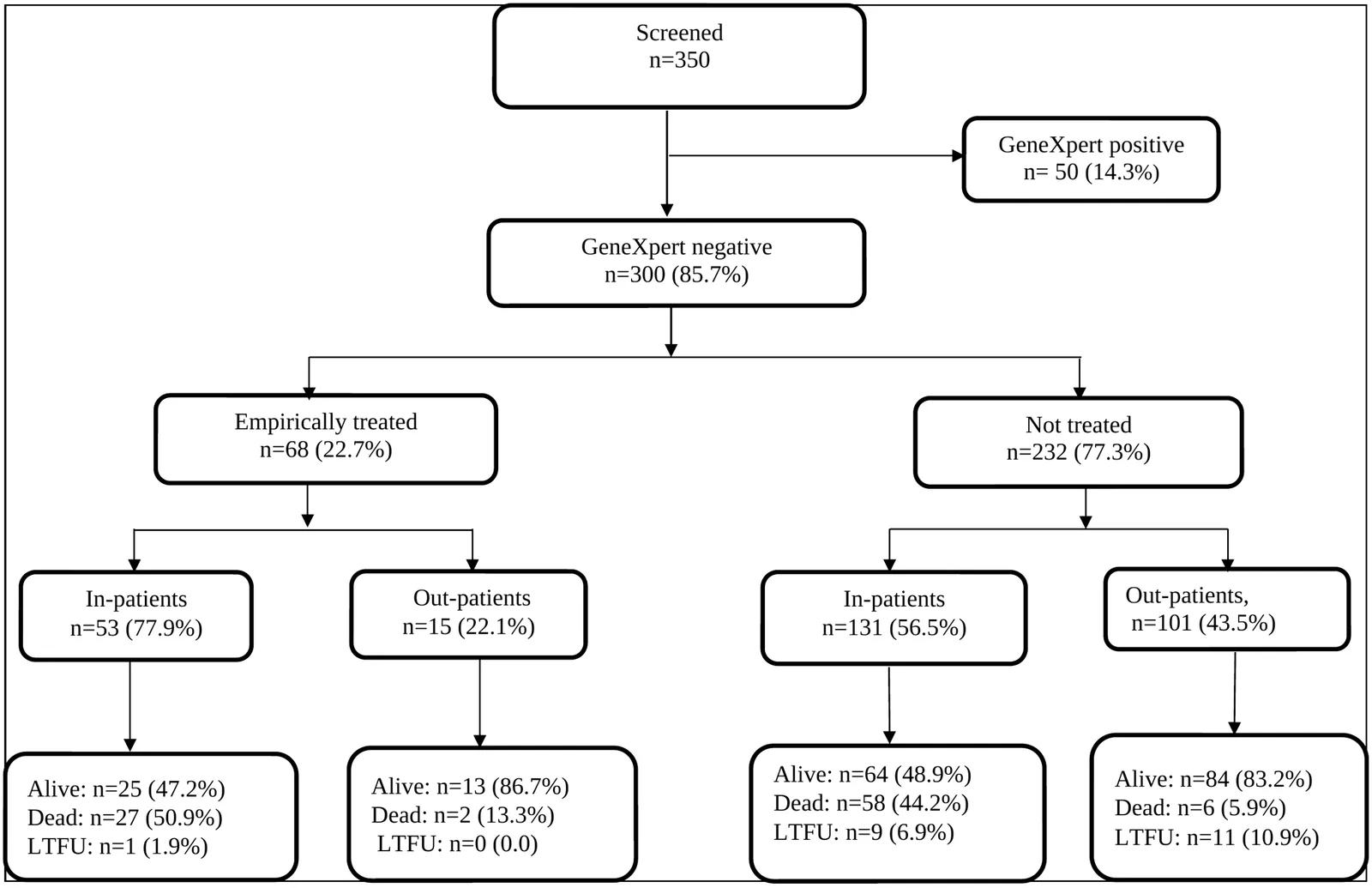
Showing flow of study participants.

Overall, 300 Xpert-negative PLHIV participants were eligible for analysis (Fig 1); 61.3% (184/300) inpatients, 55.7% (167/300) female with median age 37 (IQR 29–45) years. Majority (82.3%, 247/300) of the participants were taking ART, with median CD4 cell count 206 (IQR 36–507) cells/mm^3^. A total of 82/300 (26.7%) participants had been previously treated for drug susceptible TB (Table 1).

**Table 1:** Baseline participant characteristics stratified by empiric TB treatment status.

Characteristic	Overall (N = 300)	Empiric TB treatment (n = 68)	Not treated for TB (n = 232)	p-value
Median age, years (IQR)	37 (29–45)	35 (27–44)	38 (29–45)	0.161
Inpatients, n (%)	184 (61.3)	53 (77.9)	131 (56.5)	0.002
Female sex, n (%)	167 (55.7)	35 (51.5)	132 (56.9)	0.428
BMI, kg/m^2^, (IQR)	21.2 (18.9–24.9)	19.9 (18.0–22.5)	22.2 (18.9–24.9)	0.002
Fever (Temperature≥37.5^0^C), n (%)	26 (8.7)	10 (14.7)	16 (6.9)	0.005
Karnofsky score, % (IQR)	80 (70–80)	70 (70-80)	80 (70–80)	0.003
On ART, n (%)	247 (82.3)	50 (73.5)	197 (84.9)	0.030
Duration on ART, months (IQR)	13 (6–36)	13 (13–30)	13 (5–36)	0.353
Median CD4 count, cells/mm^3^ (IQR)	206 (36–507)	87 (16–335)	373 (158–638)	<0.001
Previous TB treatment, n (%)	82 (27.3)	20 (29.4)	62 (26.7)	0.622
CXR suggestive of TB* ^∞^ , n (%)	11 (4.7)	10/68 (14.7)	1/167 (0.59)	0.004
Positive sputum TB culture, n (%)	15 (5.0)	11 (16.2)	04 (1.7)	<0.001
MTB	11 (3.7)	8 (11.8)	3 (1.3)	_
MOTT	04 (1.3)	3 (4.4)	1 (0.4)	_
Urine TB-LAM positive, n (%)	23 (7.7)	16 (23.5)	07 (3.0)	0.011
Grade 1+	14 (4.7)	11 (16.2)	3 (1.3)	_
Grade 2+	8 (2.7)	5 (7.4)	3 (1.3)	_
Grade 3+	1 (4.6)	0	1 (0.4)	_
Positive serum CrAg^§^, n (%), N = 242	37 (15.3)	33 (48.5)	4 (2.3)	<0.001
Positive blood culture^¤^, n (%), N = 241	11 (4.6)	10 (14.7)	1 (0.6)	<0.001
Cryptococcus Neoformans	3 (1.2)	3 (4.4)	0	_
Coagulase negative staph	2 (0.8)	1 (1.5)	1 (0.6)	_
Staphylococcus Aureus	1 (0.4)	1 (1.5)	0	_
E. coli	1 (0.4)	1 (1.5)	0	_
Others (Klebsiella oxytoca, Aeromonas, Bacillus, Enterobacter species	4 (1.7)	4 (5.9)	0	_

*CXR available for 234/300 participants, 66 participants had missing CXR data (missing or not performed); ^∞^CXR interpreted by attending clinician; § CrAg results available for 242/300 participants; 58 had missing data; ^¤^Blood culture results available for 241/300 participants; 59 had missing data.

**Abbreviations:** IQR, interquartile range; CrAg, Cryptococcal Antigen; TB, tuberculosis; MTB, Mycobacterium tuberculosis; MOTT, Mycobacterial Other than Tuberculosis; ART, antiretroviral therapy; CXR, chest X-ray; LAM, lipoarabinomannan; BMI, Body Mass Index; E. coli, Escherichia coli; _, subgroup comparison not performed.

Chest X-ray (CXR) was interpreted as suggestive of TB by the attending clinician in 11 (4.7%) of the participants with CXRs available. According to patient records, the attending clinician often interpreted CXR as suggestive of TB if radiological findings such as lung consolidation, upper-lobe infiltrates, cavitation, miliary pattern were present. Fifteen (5%) participants had positive sputum TB culture, 11 of these were Mycobacterium tuberculosis complex (MTBc) while four were mycobacterium other than tuberculosis (MOTT). Urine TB-LAM was positive in 23 (7.8%) of the 300 participants, nine [9] of whom had strongly positive LAM bands (2+ and more). Serum CrAg was positive in 37, of whom 3 were also blood culture positive for Cryptococcus Neoformans. Blood cultures were positive in 8 additional participants, although 2 were likely contaminated with coagulase-negative staphylococcus. Table 1 presents participant characteristics stratified by empiric TB treatment status.

### Empiric TB treatment decision among Xpert-negative PLHIV

Overall, 68 (22.7%) of the 300 study participants received empiric TB treatment, the majority (53/68; 77.9%) coming from the inpatient setting (Fig 1). Compared to those who were not treated, participants who received empiric TB treatment were less likely taking ART at the time of enrolment (73.5% vs. 84.9%; p = 0.030), median CD4 cell counts were significantly lower (87 cells/µL vs. 373 cells/µL; p = 0.001), and had a significantly lower median body mass index (BMI) (19.9 kg/m² vs. 22.2 kg/m²; p = 0.002). Sputum TB culture was positive among 11 (8 MTBc and 3 MOTT) of those who received empiric TB treatment. The most frequent findings that could potentially have influenced the decision not to initiate empiric TB treatment by the attending clinician inferred from the patient records were; outpatient status, less severe presentation, and clinician decision to await further diagnostic results or to initiate antibiotics. A comparison of baseline demographic and clinical characteristics by empiric TB treatment decision is presented in Table 1.

### 12-months mortality among study participants

At 12 months post-enrolment, of the 300 participants, 31.0% (93/300) had died, 69 (74.2%) of these were on ART. Of the 93 deaths, 46.1% (85/184) deaths occurred among inpatients and 6.9% (8/116) among outpatients (p = 0.001). The overall total time at risk for the 93 participants who died was 300 person-months, with a mortality rate of 310 deaths per 1000 person-months. A total of 21 (7.0%) participants were lost to follow-up, while 62.0% (186/ 300) were still alive (Fig 1).

**Fig 2:**
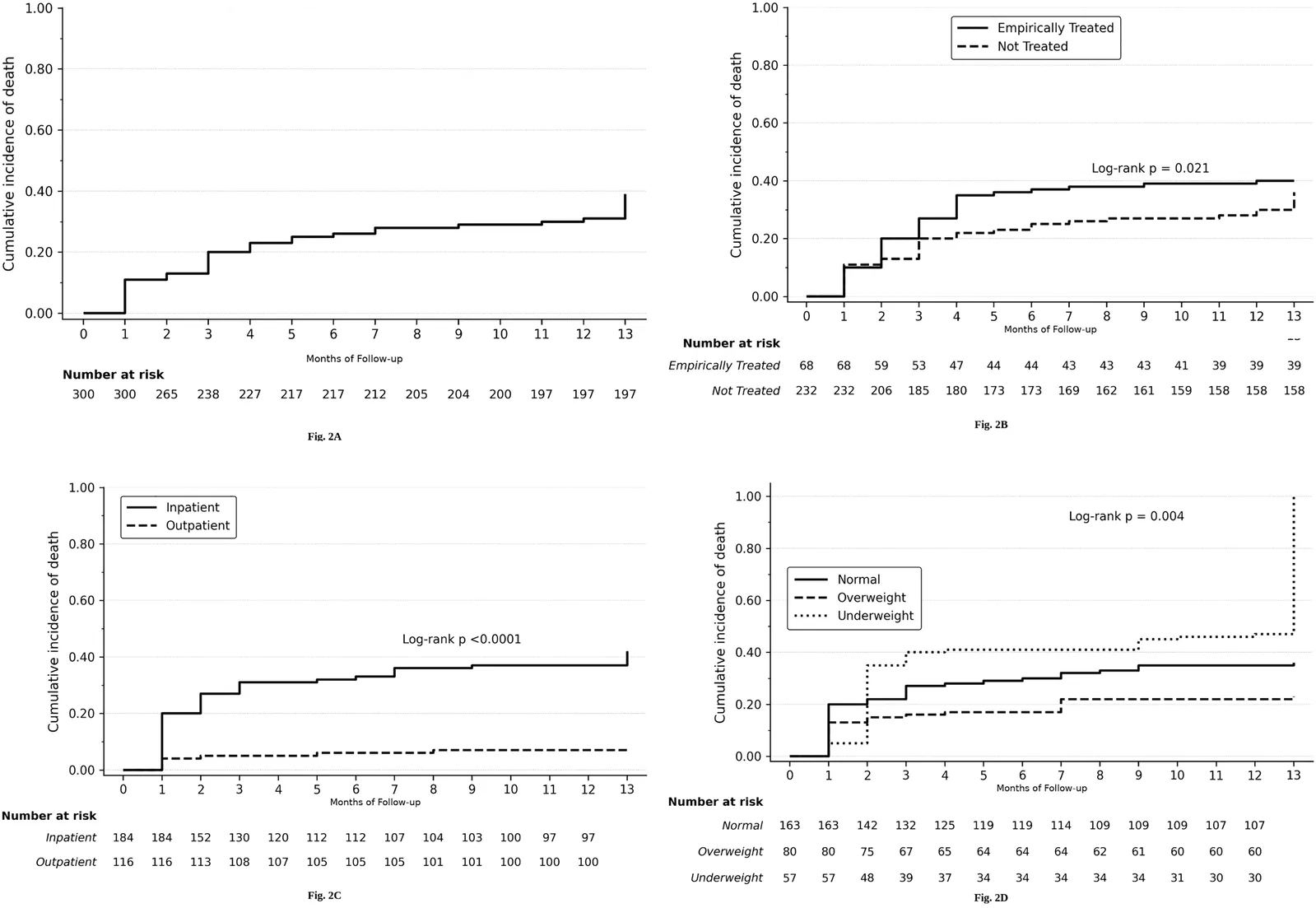
Kaplan–Meier estimates of 12-month mortality among participants. **(A)** showing Kaplan–Meier 12-month mortality estimates for overall study participants; **(B)** showing Kaplan–Meier 12-month mortality estimates by empiric TB treatment decision; **(C)** showing Kaplan–Meier 12-month mortality estimates by care setting (inpatient vs outpatient); **(D)** showing Kaplan–Meier 12-month mortality estimates by Body Mass Index.

Kaplan-Meier survival curves to estimate and compare the cumulative probability of mortality over time stratified by key variables including sex, age, empirical TB treatment status, and patient care setting (inpatient vs outpatient) are illustrated in Fig 2. Overall, mortality experience increased over the follow-up period, with approximately 31% cumulative mortality by month 12. Most deaths occurred within the first three months (Fig 2A). Mortality differed significantly by empiric TB treatment status, patient care setting and body mass index (BMI).

Participants empirically treated for TB had a significantly higher mortality compared to those not treated (log-rank p = 0.021), with mortality more pronounced in the first three months of recruitment and a gradual decrease in deaths over time (Fig 2B). Similarly, inpatients experienced a significantly higher mortality compared to out-patients (log-rank p < 0.001), with cumulative mortality exceeding 60% by month 12 (Fig 2C), and so was underweight when compared to normal body weight (p = 0.004) (Fig 2D).

### 12-months mortality and empiric TB treatment decision

Of the 93 participants who died, of whom 69 (74.2%) were taking ART at the time of enrolment, 29/93 (31.2%) had received empiric TB treatment, while 64 (69%) had not. There was a statistically significant difference in proportions of death between those who received empiric TB treatment and those who did not, that is, 42.6% of the 68 empirically treated vs 27.5% of the 232 who were not, log-rank p = 0.021, although this difference did not persist after adjusting for indicators of severe illness. Notably, 7.5% [7] and 8.6% [8] of the 93 deaths had positive TB culture and urine TB-LAM tests respectively, yet in the majority, empiric TB treatment initiation decision had not been made (Table 2). A total of 16 (17.2%) of the 93 deaths had positive serum CrAg, of which four had received empiric TB treatment. Cryptococcal treatment was not documented.

**Table 2:** Laboratory results of the 93 participants who died.

	Overall deaths, (N = 93)	Empiric TB, Treatment, N = 29	Not treated, N = 64
Positive urine TB-LAM, n (%)	8 (8.6)	2 (6.9)	6 (9.4)
Positive sputum MTB cultures, n (%)	07 (7.5)	3 (10.3)	4 (6.3)
Positive serum CrAg, n (%)	16 (17.2)	4 (13.8)	12 (18.8)
Positive blood cultures, n (%)	7 (7.5)	1 (3.4)	6 (9.4)

N is the total number of deaths, n = number of dead participants with the specified laboratory result.

**Abbreviations:** TB, tuberculosis; TB-LAM, lipoarabinomannan; CrAg, cryptococcal Antigen; TB, tuberculosis; MTB, Mycobacterium tuberculosis.

### Mortality risk among study participants

Mortality risk ratios and mortality rates are presented in Table 3. Notably, compared to those not treated, participants empirically treated for TB had a higher mortality rate (51.51, 95% CI: 35.80–74.12 vs 30.15, 95% CI: 23.60–38.52 per 1,000 person-months), as well as a 55% increased risk of death (Risk Ratio, RR; 1.55; 95% CI: 1.09–2.18; p = 0.013). Inpatients had a higher mortality rate (59.78 per 1,000 person-months; 95% CI: 48.33–73.93) compared to outpatients (6.32 per 1,000 person-months; 95% CI: 3.17–12.66), while participants on ART had lower mortality rate compared to those not on ART (29.96, 95% CI 23.66–37.93 vs 62.66, 95% CI: 42.00–93.49 per 1,000 person-months) (Table 3).

**Table 3:** Mortality risk and rates for the 300 Xpert-negative PLHIV participants.

Characteristic	n/N	Mortality Risk Ratio (95%CI)	P-value	Mortality rate per 1000 person-months (95% CI)
**Sex**				
Male	47/133	1 (ref)		41.16 (30.92–54.78)
Female	46/167	0.78 (0.56–1.09)	0.147	29.79 (22.32–39.78)
**Age (years)**				
< 40	53/165	1 (ref)		36.63 (27.98–47.94)
≥ 40	40/135	0.92 (0.66–1.30)	0.643	32.28 (23.68–44.01)
**Empirically treated for TB**				
No	64/232	1 (ref)		30.15 (23.60–38.52)
Yes	29/68	1.55 (1.09–2.18)	0.013	51.51 (35.80–74.12)
**Patient care setting**				
Out-patient	8/116	1 (ref)		6.32 (3.17–12.66)
In-patient	85/184	10.5 (4,5–24.3)	<0.001	59.78 (48.33–73.93)
**On ART**				
No	24/53	1 (ref)		62.66 (42.00–93.49)
Yes	69/247	0.62 (0.43–0.88)	0.008	29.96 (23.66–37.93)
**Previous TB treatment**				
No	69/211	1 (ref)		37.66 (29.75–47.69)
Yes	22/80	0.85 (0.56–1.26)	0.402	28.76 (18.94–43.68)
**Body Mass Index**				
Normal	54/163	1 (ref)		37.04 (28.37–48.36)
Underweight	25/57	1.22 (0.81–1.85)	0.133	56.43 (38.13–83.52)
Overweight/Obese	14/80	0.40 (0.23–0.70)	0.002	17.83 (10.56–30.11)
**Karnofsky score**				
≥ 80	17/157	1 (ref)		10.19 (6.33–16.38)
< 80	76/143	4.92 (3.05–7.89)	<0.001	74.73 (59.68–93.57)

N is total number of participants in the subgroup; n is the number of deaths in the subgroup.

**Abbreviations**: Ref, reference category; BMI, body mass index; ART, antiretroviral therapy; CI, confidence interval; TB, tuberculosis.

### Factors associated with 12-month cumulative mortality among participants

Table 4 presents crude and adjusted mortality rate ratios (MRRs) estimated using Poisson regression models with robust standard errors. In the bivariate analysis, empiric TB treatment (42.7% mortality; crude MRR 1.71; 95% CI 1.05–2.76; p = 0.029), inpatient care (46.3% mortality; crude MRR 9.84; 95% CI 4.66–20.78; p < 0.001), not taking ART (46.6% mortality; crude MRR 2.08; 95% CI 1.22–3.55; p = 0.008), and poor functional status (Karnofsky score <80; 52.9% mortality; crude MRR 7.87; 95% CI 4.42–14.00; p < 0.001) were each associated with higher 12-month mortality. In the multivariable model, adjusted for inpatient status, ART use, BMI, KPS, age sex and previous TB treatment, empiric TB treatment was no longer significantly associated with mortality (adjusted MRR 1.05; 95% CI: 0.62–1.77; p = 0.850). Only inpatient status (adjusted MRR 3.80; 95% CI 1.42–10.16; p = 0.008) and poor functional status (Karnofsky score <80; adjusted MRR 3.50; 95% CI 1.69–7.23; p = 0.001) remained independently associated with mortality. Other patient characteristics, including ART status, sex, age group, previous TB treatment, and BMI, were not significantly associated with mortality in the adjusted model (Table 4).

**Table 4:** Factors associated with 12-month mortality among Xpert-negative PLHIV: crude and adjusted Poisson regression.

Characteristics		Univariate Analysis		Multivariate Analysis	
	Mortality rate at 12 months (%)	Crude MRR (95% CI)	P-Value	Adjusted MRR (95% CI)	P-Value
**Sex**					
Male	36.0 (28.5–44.9)	1(ref)		1	
Female	27.2 (21.1–34.7)	0.72 (0.47–1.12)	0.15	0.79 (0.48–1.28)	0.336
**Age (years)**					
< 40	32.1 (25.5–39.9)	1 (ref)		1	
≥ 40	29.9 (22.9–37.8)	088 (0.57–1.37)	0.577	0.82 (0.50–1.34)	0.427
**Empirically treated**					
No	27.6 (22.3–33.9)	1 (ref)		1	
Yes	42.7 (32.0–55.4)	1.71 (1.05–2.76)	0.029	1.05 (0.62–1.77)	0.85
**Patient category**					
Out-patient	6.9 (3.5–13.4)	1 (ref)		1	
In-patient	46.3 (39.4–53.9)	9.84 (4.66–20.78)	<0.001	**3.80 (1.42–10.16)**	**0.008**
**On ART**					
No	46.6 (34.1–61.1)	1 (ref)		1	
Yes	27.9 (22.7–34.0)	0.48 (0.28–0.81)	0.008	0.66 (0.36–1.19)	0.167
**Previous TB treatment**					
No	33.3 (27.4–40.3)	1 (ref)		1	
Yes	26.3 (18.0–37.5)	1.71 (1.06–2.76)	0.029	1.11 (0.64–1.92)	0.173
**Body Mass Index**					
Normal	33.4 (26.7–41.3)	1 (ref)		1	
Underweight	42.6 (30.9–56.6)	1.52 (0.90–2.57)	0.116	1.47 (0.85–2.55)	0.173
Overweight/Obese	17.9 (11.0–28.3)	0.47 (0.24–0.90)	0.002	0.83 (0.38–1.79)	0.117
**Karnofsky score**					
≥ 80	11.1 (7.0–17.2)	1 (ref)		1	
< 80	52.9 (44.9–61.3)	7.34 (4.23–12.72)	<0.001	**3.50 (1.69–7.23)**	**0.001**

**Abbreviations:** TB, tuberculosis; ART, antiretroviral therapy; Ref, reference category; CI, confidence interval; MRR, mortality rate ratio

## Discussion

We found high 12-month cumulative mortality among Xpert-negative presumptive TB adult PLHIV, mostly occurring in the first three months after enrolment and predominantly among inpatient participants. Although crude mortality was higher among those who received empiric TB therapy compared to those who did not, this association was no longer significant after adjustment for severe illness indicators, particularly inpatient and functional status, suggesting confounding by indication. Participants who received empiric treatment were sicker at baseline (lower CD4 counts, lower BMI, higher inpatient proportion), and the higher crude mortality compared to the non-treated largely reflects underlying baseline disease severity rather than a causal effect of empiric treatment. Inpatient status and low Karnofsky Performance Score (KPS) were independent predictors of 12-month mortality. Our study also demonstrated that Cryptococcosis and bacteremia were not uncommon, highlighting the existence of concurrent non-TB conditions in this patient population.

We report 31% cumulative mortality among Xpert-negative presumptive TB PLHIV, despite majority taking ART. This is consistent with earlier studies that have evaluated mortality in similar populations. In 2017, Heunis et al in South Africa reported a 36.7% mortality among HIV/TB co-infected patients, and 23.8% among those who were smear-negative and had missed early TB diagnosis due to diagnostic challenges [32]. The South African study was a 10-year retrospective records review of risk factors for TB mortality in the general population and did not include Xpert-negatives. Similar trends have been observed in Brazil, where late TB diagnosis in PLHIV, particularly those with negative Xpert results, has been associated with delayed treatment and poor outcomes [33].

Mortality in Xpert-negative adult PLHIV may be due to either missed TB diagnosis or misdiagnosis of concurrent non-TB conditions. Studies have shown that smear-negative patients may have other pulmonary or systemic diseases that mimic TB including bacterial pneumonia, pneumocystis jirovecii pneumonia, chronic pulmonary aspergillosis and malignancies, all of which are recognized contributors to poor outcomes and mortality in PLHIV [12,34]. Misdiagnosis or failure to recognize these conditions can delay appropriate treatment, contributing to mortality. Inadequate diagnostics in RLS further exacerbate these challenges [35,36]. In sub-Saharan Africa (SSA) and other low-income regions, access to comprehensive diagnostic tools, like Xpert Ultra, chest Computed Tomography (CT) scans or bronchoscopy is limited [5], often resulting in empiric treatment based on clinical suspicion rather than confirmed diagnosis [37].

Our study observed higher unadjusted mortality among patients who received empiric TB treatment. This finding contrasts with the study by Huerga et al. (2019) [8], which reported no association between TB treatment and increased mortality among PLHIV, and another 8-week follow-up study which found reduced risk of mortality at 8 weeks of empiric treatment in a population of smear-negative PLHIV [19]. Whereas Huerga et al. evaluated six-month mortality among all patients initiated on TB treatment regardless of whether treatment was empiric or based on bacteriological confirmation, our study was restricted to Xpert-negative PLHIV. This narrower focus may reflect a subgroup with more advanced disease, greater diagnostic uncertainty, and higher baseline mortality risk, potentially accounting for the higher mortality observed in our study. The longer duration of follow-up (up to 12 months) in our study also explains the higher absolute mortality, although majority of our participants died within the first three months.

Nevertheless, our findings are consistent with a study from Brazil, which reported higher mortality among PLHIV with negative sputum smear who received empiric TB treatment [33]. Similarly, a Ugandan cohort study found higher unadjusted mortality among smear-negative PLHIV initiated on empiric TB treatment after one-year of follow-up; however, this association was no longer significant after adjustment for CD4 cell count and ART use [38].

An important consideration in interpreting these findings is the potential for confounding by indication. Compared to those who were not treated, participants who received empiric TB treatment were clinically more severely sick at baseline. Specifically, participants who received empiric TB treatment had lower CD4 counts, lower body mass index (BMI), poorer functional status (KPS < 80%), and were more likely to be hospitalized. These features suggest that clinicians prioritized empiric TB treatment among sicker patients in the setting of diagnostic uncertainty, an indication of appropriate clinical decision-making rather than a harmful effect of empiric TB treatment. After adjusting for the baseline clinical differences indicating severe illness, empiric TB treatment was no longer significantly associated with increased mortality, which strongly suggests confounding by indication. This finding provides assurance that empiric TB treatment, when clinically indicated, may not independently worsen patient outcomes or cause increased mortality. Our findings are consistent with the earlier clinical trials REMEMBER [22] and STATIS [26] which found no mortality benefit or harm from empiric TB treatment in ART naïve PLHIV with advanced disease. Our findings extend these earlier observations from clinical trials to a real-world setting with widespread ART coverage. It should be noted that unlike the earlier trials, majority of participants in our study were taking or had been on ART.

Mortality in our cohort occurred predominantly within the first three months post-enrolment and was concentrated among hospitalized patients. The observed early mortality in our study is of clinical significance and may indicate late presentation to care, delayed diagnosis, or missed alternative diagnoses rather than failure of TB treatment alone. This timing corresponds to the intensive phase of TB treatment, during which PLHIV, particularly those with advanced HIV disease remain highly vulnerable to complications including TB drug toxicity and unmasked opportunistic infections. Although the absence of viral load monitoring in our study limits our ability to confirm these mechanisms and the presence of advanced HIV disease, inpatient status and low KPS, both indicators of underlying severe illness, were found to be independent predictors of mortality. These findings suggest that baseline disease severity, rather than empiric TB treatment itself, was the main driver of mortality in this population.

The finding that 15.6% of non-treated participants who died had either a positive TB culture and/or positive urine TB-LAM further suggests that some cases of paucibacillary TB were missed by Xpert in some participants, potentially contributing to mortality in the absence of appropriate TB treatment. This highlights the diagnostic challenges in detecting paucibacillary TB among PLHIV particularly with advanced disease, where even sensitive molecular tests like Xpert have reduced sensitivity due to low bacillary burden [5,11,16]. This finding highlights the need to scale-up and prioritize more sensitive TB tests particularly Xpert Ultra [13,16] at health facilities serving PLHIV in Uganda and similar high burden TB/HIV settings. Additional diagnostic approaches including TB culture should also be more widely available in high HIV/TB prevalence settings to improve TB detection among PLHIV. It should be noted that TB culture and urine TB LAM investigations were not routine at the time of the study, and thus these patients were considered non-TB by the attending clinician.

We also investigated possible non-TB conditions that could occur either as comorbidities or alternative diagnoses in this population of X-pert negative PLHIV. We found a high frequency of cryptococcal antigenemia and bacterial blood stream infections including Staphylococcus aureus, E.coli among others, which may have contributed to the high mortality observed in our cohort. It is also possible that once empiric TB treatment is initiated, clinicians are less likely to pursue further investigations for other possible concurrent non-TB conditions, which could potentially explain the high mortality observed in our study. These findings highlight the need for comprehensive diagnostic evaluation for comorbidities and alternative disease conditions both before and alongside empiric TB treatment in PLHIV.

Among participants found with cryptococcosis, nearly 90% were empirically treated for TB, yet more than 40% died, highlighting how delayed or missed diagnosis of opportunistic infections may significantly contribute to mortality in advanced HIV disease despite empiric TB treatment [22,25,26]. In Uganda, disseminated cryptococcosis and cryptococcal meningitis remain major causes of death among PLHIV with advanced disease [39], emphasizing the importance of routine screening for these common opportunistic infections. Other HIV-associated conditions such as Kaposi’s Sarcoma and HIV-associated lymphoma can also clinically and radiologically mimic TB. An earlier report from Uganda showed that lymphoma is frequently initially misdiagnosed as TB at the Uganda Cancer Institute [38], emphasizing the need for broader diagnostic evaluation in PLHIV with presumed TB. More advanced imaging modalities like Computed Tomography (CT) scan could detect disease conditions radiologically mimicking TB such as aspergillosis, malignancy, Pneumocystis pneumonia, that are not easily detectable on CXR, thereby reducing reliance on empiric TB treatment.

Our findings have important implications for clinical practice and policy in Uganda and similar high burden TB/HIV settings. In PLHIV with negative Xpert, clinicians should perform a comprehensive evaluation for comorbidities and alternative non-TB conditions including cryptococcal infection, blood stream infections and other opportunistic infections, which occur commonly among PLHIV before and during any empiric TB treatment decision. Additionally, the high inpatient mortality particularly within the first three months highlights the need for rapid and extensive clinical assessment, early initiation of appropriate treatment, and follow-up of hospitalized PLHIV with presumptive TB and negative Xpert results. Our findings support the World Health Organization recommendations advocating for parallel evaluation for TB and HIV-related conditions, rather than a sequential diagnostic approach in PLHIV with advanced disease.

We acknowledge some study limitations. First, this was an observational study, and despite multivariable adjustment for key confounders, residual confounding by indication cannot be excluded. Participants who received empiric treatment were considerably sicker at baseline, and unmeasured confounders such as HIV viral load, co-morbidities, and nutritional status beyond what we have presented may have contributed to the observed mortality differences. Additionally, viral load data were unavailable as part of the study, which also limited assessment of HIV control as a potential confounder. Secondly, the study did not establish causes of death during the telephone follow-up, and no post-mortem data were available. The inability to categorize causes of death limits interpretation of the specific mechanisms driving mortality in each group. Thirdly, TB treatment adherence and discontinuation data were not collected at individual level, and yet, interruptions to treatment if present could influence outcomes. However, since all participants were managed at the respective health facilities under the National TB program, the study relied on patient adherence information routinely collected. Routine adherence monitoring is by clinical reviews and pill counts, and any adherence issues are handled at the clinic in real-time. Fourth, monitoring of CD4 cell count trends and evaluation for infections post-enrolment was not performed, limiting our ability to prognosticate patient outcomes. Missing data for some variables such as CXR, CrAg and blood culture may also have attenuated the strength of the association. Lastly, the loss to follow-up in this study was 7%, and although minimal, it could have introduced bias into the mortality estimates, particularly for the participants in whom empiric TB treatment was not considered.

## Conclusion

In this high-burden TB/HIV setting, we found high mortality among Xpert-negative presumptive TB adult PLHIV, occurring predominantly among hospitalized individuals and within the first three months post-enrolment. Although unadjusted mortality was higher among participants who received empiric TB treatment, this association was lost after adjustment for baseline characteristics indicative of severe illness. Inpatient status and poor functional status were independent predictors of 12-month mortality among Xpert-negative PLHIV.

Our findings do not argue against empiric TB treatment in PLHIV with Xpert-negative tests where clinical judgement remains essential. Rather, they highlight the need to expand and improve access to investigations for non-TB comorbid conditions, particularly cryptococcal disease and bacteremia, which are not uncommon in this population. The investigations should be performed before and alongside empiric TB treatment. Expanding access to diagnostics including TB culture, Xpert Ultra, bacterial cultures, and tests for the common opportunistic infections alongside adequate follow-up systems is essential to mitigate mortality in Xpert-negative PLHIV.

## Supporting information

S1 Data Empiric TB dataset Uganda. (DTA)
